# A Novel *SERPINB1* Single-Nucleotide Polymorphism Associated With Glycemic Control and β-Cell Function in Egyptian Type 2 Diabetic Patients

**DOI:** 10.3389/fendo.2020.00450

**Published:** 2020-07-30

**Authors:** Dina H. Kassem, Aya Adel, Ghada H. Sayed, Mohamed M. Kamal

**Affiliations:** ^1^Department of Biochemistry, Faculty of Pharmacy, Ain Shams University, Cairo, Egypt; ^2^Pharmacology and Biochemistry Department, Faculty of Pharmacy, The British University in Egypt, Cairo, Egypt; ^3^The Center for Drug Research and Development (CDRD), Faculty of Pharmacy, The British University in Egypt, Cairo, Egypt; ^4^Department of Clinical and Chemical Pathology, National Institute of Diabetes & Endocrinology, Cairo, Egypt

**Keywords:** serpinB1, type 2 diabetes mellitus, β-cell dysfunction, insulin resistance, hepatokines, gene polymorphism

## Abstract

**Aims:** Serine protease inhibitor B1 (SerpinB1) is a neutrophil elastase inhibitor that has been proved to be associated with type 2 diabetes mellitus and pancreatic β-cell proliferation. In this study, we investigated 2 *SERPINB1* SNPs, rs114597282 and rs15286, regarding their association with diabetes risk and various anthropometric and biochemical parameters in Egyptian type 2 diabetic patients.

**Materials and Methods:** A total of 160 subjects (62 control and 98 type 2 diabetic patients) participated in this study. Various anthropometric and biochemical parameters were assessed. Genotyping assay for the two SNPs was done using TaqMan genotyping assays. The association of rs15286 variants with risk of diabetes, various biochemical parameters, and glycemic control in diabetic patients was assessed.

**Results:** All genotyped subjects were found to be homozygous TT for *SERPINB1* rs114597282. All genotype variants of *SERPINB1* rs15286 were found in our Egyptian subjects with A being the minor allele. The SNP rs15286 was not found to be associated with risk of diabetes. The AA genotype was found to be associated with lower fasting plasma glucose, lower HbA_1c_%, and better β-cell function and glycemic control in diabetic patients. The G allele was associated with poor glycemic control.

**Conclusions:** The genotypes AA, AG, and GG of *SERPINB1* gene SNP rs15286 are all represented in the studied sample; however, it is not associated with risk of diabetes. Genotype AA of SNP rs15286 is associated with better glycemic control and better β-cell function in diabetic patients, while the G allele potentially represents the “risk allele” of poor glycemic control.

## Introduction

Diabetes mellitus (DM) is a complex multifaceted metabolic disorder. Unfortunately, its global prevalence is growing at an alarming rate especially in middle- and low-income countries (1). The number of people suffering DM globally has risen from about 108 million in 1980 to nearly 422 million in 2014 (2), with a further expected increase to about 630 million people worldwide by the year 2045 (1). Type 2 DM is the most common type of DM, and genetic-predisposition accounts for nearly 60–90% of the susceptibility to its development (3). During the natural course of the disease, insulin resistance and β-cell loss or dysfunction are potential drivers for the various metabolic abnormalities associated with type 2 DM (3, 4). When the functional insulin-secreting β-cell mass is compromised, the normal physiological glucose homeostasis is disrupted and type 2 DM manifests (5).

Accordingly, vigorous efforts have been exerted over the past years to develop strategies which would help to compensate and/or expand functional β-cells. Among those approaches was the identification of factors/mediators capable of inducing proliferation and expansion of preexisting functional β-cells (5, 6). That approach attracted much interest especially that β-cell expansion and compensation capabilities have been reported in various conditions associated with insulin resistance such as obesity (7, 8) or pregnancy (9). Interestingly, in this regard, *El-Ouaamari and coworkers* highlighted the integrative cross talk between the liver and pancreatic β-cells via the secretion of hepatocyte-derived factor(s) in response to insulin resistance which induces β-cell proliferation (10).

Among these hepatocyte-derived factors, serine protease inhibitor B1 (serpinB1; serpin family B member 1) has been identified to play an important role in that process of β-cell compensation in response to insulin resistance (11). Interestingly, serpinB1 was also previously reported to act as a neutrophil elastase inhibitor (12), and improving glucose tolerance and insulin sensitivity was found to be associated with such inhibition (13). Additionally, a recent report sheds light on the possible association of serpinB1 with insulin sensitivity in healthy adults (14). It is noteworthy that serpinB1 is not the only serpin which could be associated with insulin sensitivity. Long before, the visceral adipose tissue-derived serpin—Vaspin—was also identified as an interesting insulin-sensitizing adipokine (15), with a putative interplay with other mediators in compensatory mechanisms for insulin resistance in type 2 DM (16).

Nevertheless, genetic variants of the *SERPINB1* gene and their possible implication into β-cell dysfunction and reduced β-cell compensation in diabetic patients have not been investigated (17). Just one family has been identified with a possibly damaging *SERPINB1* variant associated with diabetes (11). Thus, knowing that genome-wide association studies have revealed several genetic variants related to compromised β-cell function to be associated with type 2 DM (18, 19), as well as the extra layer of complexity in different ethnic populations (20), this inspired us to investigate if genetic variants of *SERPINB1* are associated with diabetes risk, glycemic control, and β-cell dysfunction in Egyptian type 2 diabetic patients. According to our knowledge, this is the first report investigating the association of *SERPINB1* genetic variants with type 2 DM.

## Materials and Methods

### Study Population and Anthropometric Measurements

This study was approved by the ethical committee of the National Institute of Diabetes and Endocrinology—General Organization for Teaching Hospitals and Institutes (NIDE—GOTHI) under number IDE00203, and informed consent was obtained from every enrolled subject. The study was carried out in accordance with the Declaration of Helsinki recommendations and regulations (21). A total of 160 subjects were enrolled in the study; 62 non-diabetic healthy control subjects and 98 patients with type 2 DM. The definition of a non-diabetic was a subject who has a fasting plasma glucose (FPG) level lower than 110 mg/dl and has no family history of type 2 DM. All the control subjects were not receiving any dietary supplements or medications and were not suffering any health problems. The 98 patients with type 2 DM were recruited from the outpatient clinic of the National Institute of Diabetes and Endocrinology (NIDE). These 98 patients were further classified into diabetics with good glycemic control (HbA1c ≤ 7%) and diabetics with poor glycemic control (HbA1c > 7%) as described previously (22) for odds ratio calculations. The characteristics of all the study subjects are summarized in Table 1. The exclusion criteria included renal or hepatic disease, thyroid dysfunction, acute or chronic inflammatory disease, type 1 DM, ischemic cardiovascular disease, cancer, acute or chronic infections, alcohol or drug abuse, and any hematological disorder. Subjects taking hormonal therapy were also excluded.

**Table 1:** Clinical and laboratory characteristics of the studied groups.

**Factor**	**Controls**	**Type 2 DM**	***p-*value**	***p-*value^¶^**
*N* (F/M)	62 (42/20)	98 (46/52)	**……**	**……**
Age (year)	38.2 ± 1.1	49.1 ± 1.1	***<0.001^a^***	**……**.
Diabetes duration (year)	…….	8.39 ± 0.74	**……**	**……**.
BMI (kg/m^2^)	28.8 ± 0.62	31.5 ± 0.53	***0.001^a^***	***0.002***
WHR^c^	0.92 ± 0.08	0.95 ± 0.013	*0.085**^b^***	*0.845*
FPG (mg/dl)^c^	86.5 ± 1.6	209.6 ± 7.4	***<0.001^b^***	***<0.001***
HbA_1C_ (%)	5.4 ± 0.07	8.4 ± 0.22	***<0.001^a^***	***<0.001***
TC (mg/dl)	150.6 ± 5.0	209.0 ± 5.2	***<0.001^a^***	***<0.001***
LDL-C (mg/dl)	88.2 ± 5.02	127.4 ± 4.4	***<0.001^b^***	***<0.001***
HDL-C (mg/dl)^c^	47.1 ± 0.74	44.7 ± 1.41	***0.014^b^***	*0.165*
TG (mg/dl)^c^	80.2 ± 4.8	177.9 ± 9.6	***<0.001^b^***	***<0.001***
LDL-C/HDL-C^c^	1.96 ± 0.13	3.22 ± 0.19	***<0.001^b^***	***<0.001***
TC/HDL-C^c^	3.3 ± 0.15	5.11 ± 0.22	***<0.001^a^***	***<0.001***
C-peptide (ng/ml)^c^	3.81 ± 0.26	0.84 ± 0.054	***<0.001^b^***	***<0.001***
HOMA2-β%^c^	213.6 ± 11.3	17.9 ± 1.2	***<0.001^b^***	***<0.001***
HOMA2-IR^c^	2.73 ± 0.19	0.94 ± 0.07	***<0.001^b^***	***<0.001***
Type of treatment (OHA/OHA + insulin/insulin)	…….	35/3/60	**……**.	**……**.

*Results are expressed as mean ± S.E.M. DM, diabetes mellitus; BMI, body mass index; WHR, waist to hip ratio; FPG, fasting plasma glucose; TC, total cholesterol; LDL-C, low-density lipoprotein cholesterol; HDL-C, high-density lipoprotein cholesterol; TG, triglycerides; HbA_1C_%, glycosylated hemoglobin; HOMA2-β%, homeostasis model assessment-β-cell function; HOMA2-IR, homeostasis model assessment-insulin resistance; OHA, oral hypoglycemic agent*.

a *Independent-sample T-test, two-tailed, p-value >0.05 non-significant*.

b *Mann–Whitney U test, two-tailed, p-value >0.05 non-significant*.

c *Log transformed for performing GLM*.

¶ *P-value after adjustment for age, gender, and BMI by GLM. All significant p-values are written in bold and italics*.

All the study subjects underwent physical examination and detailed history and laboratory investigations. Anthropometric measures included waist-to-hip ratio (WHR) and body mass index (BMI); body weight and standing height were measured in light clothing without shoes. The BMI was calculated as weight divided by squared height (kg/m^2^). Waist circumference was measured to the nearest 0.1 cm at the narrowest point between the lowest rib and the uppermost lateral border of the iliac crest, while the hip was measured at its widest point.

### Blood Sampling

All the blood samples were drawn after overnight fasting, and the samples were divided into four aliquots. The first aliquot of blood was collected on plain vacutainer tubes for serum preparation used for the assay of the lipid profile, as well as C-peptide levels. The second aliquot of blood was collected on vacutainer tubes containing sodium fluoride for measuring fasting plasma glucose (FPG). The third and fourth aliquots of blood were collected on vacutainer tubes containing sodium EDTA for measuring glycated hemoglobin (HbA_1C_%), and for subsequent DNA extraction from whole blood. Afterward, serum samples were divided into aliquots and stored at −80°C for subsequent assays.

### Laboratory Analyses

FPG and serum biochemical parameters including triglycerides (TG), total cholesterol (TC), and high-density lipoprotein-cholesterol (HDL-C) were measured using Spectrum Diagnostics kits (Egypt). Low-density lipoprotein cholesterol (LDL-C) levels were calculated by Friedewald's equation (23). HbA_1C_% was determined using ion-exchange high-performance liquid chromatography (HPLC) by the Bio-Rad D-10 system (Bio-Rad Laboratories, Hercules, CA, USA).

Afterward, serum C-peptide levels were determined using the human C-peptide ELISA kit (DRG, USA). The homeostasis model assessment of β-cell function (HOMA2-β%) and insulin resistance (HOMA2-IR) was calculated from FPG (mg/dl) and fasting C-peptide (ng/ml) levels using an online HOMA2 calculator/algorithm (24). HOMA2 models were calculated using C-peptide to avoid interference of insulin in patients treated by insulin. All ELISA procedures were done according to the manufacturer's instructions using the ChroMate microplate reader (Awareness Technology, USA).

### DNA Extraction and Genotyping Assay

The extraction of DNA from 150 μL whole blood (collected on EDTA anticoagulant) was done using the commercially available Quick DNA Miniprep Kit (Zymo Research, USA) according to the manufacturer's instructions, then the extracted DNA was quantified using a Quawell micro-volume spectrophotometer (USA).

Genotyping was done for 2 *SERPINB1* polymorphism, namely, *SERPINB1* rs114597282, which is a missense mutation with a C/T substitution (previously determined as a possibly damaging variant for *SERPINB1* gene) (11). The other SNP was *SERPINB1* rs15286, which is a transition A/G SNP (in the 3′UTR region of *SERPINB1*). Genotyping was done using TaqMan® SNP Genotyping assays with the following IDs: C_151309206_10 for rs114597282 and C____950920_1 for rs15286 using the TaqMan Universal Master Mix No UNG (Thermo Fisher Scientific, USA). Genotyping was done using a StepOnePlus thermal cycler (Applied Biosystems, USA). 20 ng of genomic DNA for each sample was genotyped using 10 μL (2×) TaqMan® Universal Master Mix, 0.5 μL (40×) TaqMan® SNP genotyping assay, and DNAse/RNAse-Free water (Gibco, Life Technologies, USA) to a total volume of 20 μL reaction using default genotyping settings with appropriate negative control.

### Statistical Analysis

All results were expressed as mean ± standard error of mean (S.E.M). The Kolmogorov–Smirnov test was done to evaluate the distribution of various variables. The independent-sample *t*-test and Mann–Whitney *U*-test were used appropriately according to the data distribution for comparison between non-diabetic control and type 2 DM groups. The genotype distribution was validated to follow the Hardy–Weinberg equilibrium (HWE) using an online calculator (25), and the chi-square (χ2) test was used to compare allele frequency distributions of various genotypes in the studied groups. Finally, binary logistic regression analysis was used to calculate the odds ratios (ORs) and 95% confidence intervals (CIs) to investigate the possible association of rs15286 variants with type 2 DM or with glycemic control status. Kruskal–Wallis or one-way ANOVA tests were used appropriately according to the distribution of data for comparison between the levels of various parameters in various genotypes (3 groups) followed by Dunn's test for Kruskal–Wallis as multiple-comparison *post hoc* tests. General linear modeling (GLM), followed by Bonferroni *post hoc* test for multiple comparisons, was used to control for covariates such as age, gender, and BMI, and *p-values* were calculated after correction for these covariates. Any non-normally distributed data was logarithmically transformed before performing GLM. All statistical analyses were performed using Windows-based SPSS statistical package (SPSS version 17.0, SPSS Inc, Chicago, IL). *P*-values ≤ 0·05 were considered significant.

## Results

### Clinical Laboratory Data of the Study Subjects

As shown in Table 1, this study included 160 subjects; 62 subjects (42 females and 20 males) who were apparently healthy volunteers served as the control group. The type 2 DM group consisted of 98 patients (46 females and 52 males). The mean age of the control group was 38.2 ± 1.1 years, while that of the type 2 DM group was 49.1 ± 1.1 years. The duration of diabetes was 8.39 ± 0.74 years. The body mass index was significantly elevated in the type 2 DM group as compared to the control group. However, the waist–hip ratio (WHR) was almost equal in both groups. In addition, FPG, HbA_1C_%, and lipid profile including TG, TC, LDL-C, and even LDL-C/HDL-C and TC/HDL-C were all significantly elevated in type 2 DM as compared to the control group.

As for β-cell function indices, C-peptide was significantly decreased in type 2 DM patients as compared to control subjects (0.84 ± 0.054 ng/ml and 3.81 ± 0.26 ng/ml, respectively, *p* < 0.001). Moreover, HOMA2-β% was also severely diminished in type 2 diabetic patients as compared to the control group (17.9 ± 1.2% and 213.6 ± 11.3%, respectively, *p* < 0.001) (Table 1).

### Association of *SERPINB1* rs15286 Variants With Type 2 DM Risk

As for the *SERPINB1* SNP rs114597282, 100% of the subjects either control or type 2 DM were found to have a homozygous TT genotype, which unfortunately hindered further processing or studying of any association of such SNP.

The distribution and the alleles' frequencies of the rs15286 SNP of *SERPINB1* are shown in Table 2. The observed distribution frequency of various alleles followed the Hardy–Weinberg equilibrium. The AA genotype represents the minor genotype in both control and type 2 DM groups with 4.8 and 6.1%, respectively, with a total of 5.625% of all subjects. On the other hand, GG was the major genotype in the genotyped subjects (60% in control and 67% in type 2 DM patients) with a total of 64.375% of all subjects. As for the AG genotype, it represented 35.5% in the control group and 26% in the diabetic group with an overall 30% of the subjects. In addition, the A allele represents the minor allele with 40% in control and 33.6% in the diabetic subjects, with a total of 35.625% of the studied subjects. The 2 groups did not differ significantly regarding the distribution of various genotypes (*p-*value = 0.479) and the frequency of A allele (*p-*value = 0.324) or G allele (*p-*value = 0.731). Moreover, the various genotypes, A allele or G allele, were not found to be associated with the risk of DM (OR = 0.655, CI = 0.322–1.332, *p-*value = 0.242; OR = 0.741, CI = 0.338–1.623, *p-*value = 0.454; OR = 0.799, CI = 0.14–4.54, *p-*value = 0.800, respectively). All ORs (95% CI) were adjusted for age, gender, and BM.

**Table 2:** Association of *SERPINB1* rs15286 variants with risk of type 2 DM.

**Serpin B1 rs15286 genotypes**	**Controls *n* = 62**	**Diabetic *n* = 98**	**χ^2^**	***p-*value**	**OR (95% CI)**	***p-*value**
	***n* (%)**	***n* (%)**				
AA	3 (4.8%)	6 (6.1%)				
AG	22 (35.5%)	26 (26.5%)	1.473	0.479	0.707 (0.392–1.274)	0.249
GG	37 (59.7%)	66 (67.4%)			0.655 (0.322–1.332)	0.242^¶^
AG/AA vs. GG	25 (40.3%) vs. 37 (59.7%)	32 (33.6%) vs. 66 (66.4%)	0.974	0.324	0.718 (0.371–1.388)	0.324
					0.741 (0.338–1.623)	0.454^¶^
AG/GG vs. AA	59 (95.2%) vs. 3 (4.8%)	92 (93.9%) vs. 6 (6.1%)	0.118	0.731	0.780 (0.188–3.238)	0.732
					0.799 (0.14–4.54)	0.800^¶^
Total	62 (100%)	98 (100%)				

*A χ2 test was done for various genotypes, A allele and G allele, and diabetes status. The odds ratio (OR) was calculated using binary logistic regression using diabetes status as the dependent variable and genotype as the covariate, in addition to adjustment for age, gender and BMI as additional covariates*.

¶ *Adjusted for the effect of covariates: age, gender, and BMI*.

### Anthropometric and Biochemical Parameters' Levels in Various Genotypes of *SERPINB1* rs15286 SNP

In order to study the levels of various parameters in the genotyped samples, we compared the levels of various anthropometric and biochemical parameters among the genotype variants in both control and type 2 DM groups. As shown in Table 3, all parameters failed to reach a significant difference among AA, AG, and GG genotypes in the control group. On the other hand, in the diabetic group, these genotypes showed a significant difference for FPG, HbA_1C_%, and HOMA2-β% (*p*-values = 0.008, 0.006, and 0.004, respectively).

**Table 3:** Association of *SERPINB1* rs15286 variants with various anthropometric and clinical characteristics of the studied subjects.

**Parameter**	**SerpinB1 rs15286 genotypes**									
	**Control**					**Type 2 DM**				
	**AA**	**AG**	**GG**	***p-*value**	***p-*value*^¶^***	**AA**	**AG**	**GG**	***p-*value**	***p-*value*^¶^***
N (F/M)	3 (3/0)	22 (15/7)	37 (24/13)	—–	—*-*	6 (4/2)	26 (13/13)	66 (29/37)	—*-*	—*-*
Age (year)	29.7 ± 3.8	40.4 ± 2.1	37.6 ± 1.3	*0.116^a^*	—–	54.7 ± 4.5	47.2 ± 2	49.3 ± 1.3	*0.271^a^*	—*-*
BMI (kg/m^2^)	30.1 ± 5.2	28.3 ± 1.2	29.1 ± 0.7	*0.778^a^*	—–	31.7 ± 1.6	31.8 ± 0.9	31.4 ± 0.7	*0.934^a^*	—*-*
WHR^c^	0.89 ± 0.02	0.92 ± 0.01	0.93 ± 0.01	*0.469^b^*	*0.772*	0.97 ± 0.02	0.95 ± 0.03	0.95 ± 0.02	*0.522^b^*	*0.670*
FPG (mg/dl)^c^	81 ± 7.2	90.8 ± 2.3	84.5 ± 2.2	*0.11^b^*	*0.17*	141 ± 7.5	224.8 ± 13.8	209.8 ± 9.1	0.008^b^^,^^**^	0.018^*^
HbA_1C_(%)^c^	5.38 ± 0.02	5.6 ± 0.1	5.3 ± 0.1	*0.083^b^*	*0.109*	6.6 ± 0.3	8.97 ± 0.3	8.4 ± 0.3	0.006^b^^,^^**^	***0.055***
TG (mg/dl)^c^	78.7 ± 24	78.9 ± 8.9	81.1 ± 6	*0.733^b^*	*0.872*	248.3 ± 44.5	172.9 ± 16.9	173.5 ± 11.8	*0.258^b^*	*0.439*
TC (mg/dl)	162.7 ± 18.8	155.1 ± 5.1	147 ± 7.7	*0.65^a^*	*0.529*	212.7 ± 21.3	212.7 ± 9.1	207.3 ± 6.6	*0.889^a^*	*0.892*
LDL-C (mg/dl)	104.7 ± 16.3	92.3 ± 5.9	84.5 ± 7.5	*0.587^a^*	*0.484*	115.7 ± 12.8	128.3 ± 8.6	128.1 ± 5.5	*0.797^a^*	*0.633*
HDL-C (mg/dl)^c^	42 ± 4.6	47.1 ± 1.2	47.5 ± 0.9	*0.385^b^*	*0.128*	47 ± 3.8	45.8 ± 2.7	44.1 ± 1.8	*0.601^b^*	*0.750*
LDL-C/HDL-C^c^	2.5 ± 0.4	2 ± 0.17	1.9 ± 0.2	*0.164^b^*	*0.147*	2.5 ± 0.29	3.2 ± 0.37	3.3 ± 0.24	*0.561^b^*	*0.716*
TC/HDL-C^c^	3.9 ± 0.5	3.4 ± 0.19	3.2 ± 0.2	*0.22^b^*	*0.208*	4.6 ± 0.37	5.1 ± 0.4	5.2 ± 0.27	*0.881^b^*	*0.878*
C-peptide (ng/ml)^c^	4.4 ± 0.8	4.1 ± 0.4	3.6 ± 0.36	*0.239^b^*	*0.386*	0.98 ± 0.2	0.75 ± 0.04	0.87 ± 0.08	*0.2^b^*	*0.247*
HOMA2-β%^c^	280 ± 75.6	202.2 ± 15.8	215 ± 15.5	*0.571^b^*	*0.413*	32.8 ± 4.7	14.4 ± 1.7	18 ± 1.5	0.004^b^^,^^**^	0.004 ^**^
HOMA2-IR^c^	3 ± 0.49	3 ± 0.29	2.5 ± 0.26	*0.127^b^*	*0.30*	0.83 ± 0.16	0.86 ± 0.07	0.98 ± 0.1	*0.781^b^*	*0.963*

*Results are expressed as mean ± S.E.M. p-values are shown for ANOVA or Kruskal–Wallis test, before and also after adjustment for age, gender, and BMI by GLM. DM, diabetes mellitus; BMI, body mass index; WHR, waist to hip ratio; FPG, fasting plasma glucose; TC, total cholesterol; LDL-C, low-density lipoprotein cholesterol; HDL-C, high-density lipoprotein cholesterol; TG, triglycerides; HbA_1C_%, glycosylated hemoglobin; HOMA2-β%, homeostasis model assessment-β cell function; HOMA2-IR, insulin resistance*.

a *ANOVA, two-tailed, p-value >0.05 non-significant*.

b *Kruskal–Wallis, two-tailed, p-value >0.05 non-significant*.

c *Log transformed for performing GLM*.

¶ *P-value after adjustment for age, gender and BMI by GLM*.

* *Significant at p < 0.05 level*.

** *Significant at p < 0.01 level. All significant p-values are written in bold and italics*.

**Figure 1:**
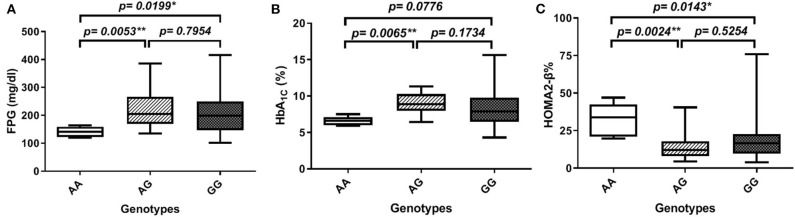
Association of *SERPINB1* rs15286 variants with various parameters in type 2 DM. **(A)** FPG, **(B)** HbA_1C_%, and **(C)** HOMA2-β%. The top and bottom whiskers represent the minimum and maximum values, while the band inside the box represents the median. *Significantly different from genotype AA at *p* < 0.05. **Significantly different from genotype AA at *p* < 0.01, assessed by the Kruskal–Wallis test followed by Dunn's *post hoc* test.

In order to gain further insight into the difference of these parameters' levels among various genotypes of diabetic patients, we compared the levels among these parameters pair-wise. Figure 1A showed that genotype AA is significantly associated with lower FPG as compared to both AG and GG genotypes (FPG AA: 141 ± 7.5 mg%, AG: 224.8 ± 13.8 mg%, and GG: 209.8 ± 9.1 mg%, *p*-value = 0.008). Similarly, HbA_1C_% was significantly lower in the AA genotype than both AG and GG genotypes but failed to reach a significant increase in GG (HbA_1C_%: AA: 6.6 ± 0.3%, AG: 8.97 ± 0.3%, and GG: 8.4 ± 0.3%, *p*-value = 0.006) as shown in Figure 1B. Interestingly, HOMA2-β% showed significant elevation in the AA genotype as compared to both AG and GG genotypes (HOMA2-β% AA: 32.8 ± 4.7%, AG: 14.4 ± 1.7, and GG: 18 ± 1.5, *p*-value = 0.004) as shown in Figure 1C. It is noteworthy that even after adjustment for age, gender, and BMI, genotype AA subjects remained relatively significantly different from the AG genotype regarding FPG, HbA_1C_%, and HOMA2-β% levels (adjusted *p*-values were 0.014, 0.057, and 0.003, respectively) and also from the GG genotype (adjusted *p-*values were 0.035, 0.23, and 0.011, respectively).

Moreover, we studied the association of the various genotypes with the glycemic control status in the genotyped diabetic patients. We found that there is a significant difference in the distribution of the various genotypes (AA, AG, and GG) between the good and the poor glycemic control of diabetic patients (*p-*value = 0.002). On the other hand, there was no significant association between good glycemic control and the AA or AG genotype, i.e., the A allele (OR = 0.627; CI = 0.233–1.691; *p-*value = 0.357). Interestingly, there was a significant association between the AA genotype and prediction of good glycemic control (OR = 10.324, CI = 1.088–97.965; *p-*value = 0.042). Actually, these last OR and CI are the same for the AG or GG genotype, i.e., the G allele and the poor glycemic control in diabetic patients. All ORs and CIs are shown before and after adjustment for age, gender, and BMI in Table 4.

**Table 4:** Association of *SERPINB1* rs15286 variants with the glycemic control status of type 2 DM.

**Serpin B1 rs15286 genotypes**	**Good glycemic control (HbA_**1C**_ ≤ 7%) *n* = 31**	**Poor glycemic control (HbA_**1C**_ >7%) *n* = 67**	**χ^2^**	***p-*value**	**OR (95% CI)**	***p-*value**
	***n* (%)**	***n* (%)**				
AA	5 (16.1%)	1 (1.5%)				
AG GG	3 (9.7%) 23 (74.2%)	23(34.3%) 43 (64.2%)	12.586	0.002^**^	0.188 (0.064–0.551) 0.212 (0.071–0.635)	0.002^**^ 0.006^¶^^,^^**^
AG/AA vs. GG	8 (25.8%) vs. 23 (74.2%)	24 (35.8%) vs. 43 (64.2%)	0.967	*0.326*	0.623 (0.242–1.606)	*0.328*
					0.627 (0.233–1.691)	*0.357^¶^*
AA vs. AG/GG	5 (16.1%) vs. 26 (83.9%)	1 (1.5%) vs. 66 (98.5%)	7.899	0.005^*^	12.692 (1.414–113.917)	0.023 ^*^
					10.324 (1.088–97.965)	0.042^¶^^,^^*^
Total	31 (100%)	67 (100%)				

*A χ2 test was done for various genotypes, A allele and G allele, and glycemic control status of diabetic patients. The odds ratio (OR) was calculated using binary logistic regression using glycemic control status as the dependent variable and genotype as the covariate, in addition to adjustment for age, gender, and BMI as additional covariates*.

¶ *Adjusted for the effect of covariates: age, gender, and BMI*.

* *Significant at p < 0.05*.

** *Significant at p < 0.01. All significant p-values are written in bold and italics*.

## Discussion

In this study, we assessed two *SERPINB1* gene SNPs in control and type 2 DM patients and investigated their association with the risk of diabetes and other anthropometric and biochemical parameters. The first SNP was *SERPINB1* rs114597282, which is a missense mutation with a C/T substitution. The other one was *SERPINB1* rs15286 which is a transition A/G SNP in the 3′UTR region of the *SERPINB1* gene. For SNP rs114597282, all our subjects, either control or diabetic patients, were of TT genotype, which did not allow any further analyses for this SNP. As for the other SNP rs15286, various genotypes, namely, AA, AG, and GG, were expressed in both control and diabetic subjects with AA being the minor genotype, while GG was the major genotype in both studied groups. However, we failed to find an association with the distribution of these genotypes with the risk of diabetes. Interestingly, we found that diabetic subjects having a rs15286 AA genotype showed lower levels of FPG and HbA_1C_%, as well as higher HOMA2-β% compared to other genotypes. Moreover, there exists a significant association of genotype AA with the prediction of good glycemic control in diabetic patients, an association not found with the A allele alone. Meanwhile, our results showed a positive association between the G allele and the prediction of poor glycemic control. This indicates that type 2 diabetic patients' carriers of the AA genotype may potentially have better control over their blood glucose levels and better β-cell function than other genotypes of this *SERPINB1* SNP. On the other hand, those who are carriers of a G allele are at risk of poor glycemic control.

SerpinB1, also known as a monocyte neutrophil elastase inhibitor, is a protease inhibitor that regulates several inflammatory responses (26). Lately, serpinB1 has been associated with insulin signaling in 2 ways. First, neutrophil elastase was found to be associated with hepatic and adipose tissue insulin resistance and its deletion may improve insulin sensitivity (13). Second, *EL-Ouaamari and coworkers* could prove serpinB1 as a novel liver-derived secretory protein that promotes proliferation of human and mouse β-cells (11). Since then, a couple of reports tried to study the possible association of serpinB1 with insulin sensitivity or with type 2 DM (14, 27). However, still the genetic variants of the *SERPINB1* gene and their association with diabetes have not been studied.

Accordingly, we decided to study two *SERPINB1* SNPs and their association with various anthropometric and biochemical parameters in type 2 DM in comparison to control subjects. One of these SNPs was introduced by EL Ouaamari et al., namely, rs114597282, as a possibly damaging variant (11). However, we failed to find the various variants in our genotyped subjects as 100% of our subjects were homozygous TT. This comes in accordance with a previous reported frequency of 1.7% among African Americans and 0.01% among Europeans according to the 1,000 Genomes database. This may well explain that we failed to get any other variants from our 160 total subjects. Accordingly, further investigations are warranted on a larger sample size and in different populations to study this SNP in association with diabetes or even other diseases.

As for the other SNP rs15286, to our knowledge, this is the first report concerning this SNP for *SERPINB1* especially in DM. Our genotyped samples showed the 3 genotypes with AA being the minor genotype representing 5.6% of total subjects, AG representing 30% of the total subjects, and GG being the major one with 64.4% of total subjects. These results approached genotype frequencies from the 1,000 Genomes database, where the overall distribution of the genotypes in all populations were AA 7% (was 5.6% in our study), AG 37% (was 30% in our study), and GG 56% (was 64.4% in our study). The A allele represents the minor allele with 36.25% in the studied groups as compared to the minor allele frequency (MAF) of 25% and the G allele of 75% from the 1,000 Genomes phase 3 database. This is the first report about the frequency of this genotype in the Egyptian population which approaches the distribution of several other populations. This SNP requires further association studies on other populations and in other diseases.

In fact, the distribution and frequency of these genotypes failed to be associated with the risk of diabetes. However, we found that the AA genotype was significantly associated with lower FPG and HbA_1C_% in diabetic patients. This finding implies that although this SNP genotype is not predictive of developing diabetes, individuals with AA genotypes are potentially less hyperglycemic and exhibit easier control on their diabetes, This was further confirmed with the positive association of the AA genotype and the good glycemic control in diabetic patients, while carriers of one G allele are under risk of poor glycemic control. We also observed that the presence of one A allele is not enough to reach a significant association with such good glycemic control. These findings may prove important in several ways. First, further studies should be conducted to explore if the patients with the AA genotype are less prone or, alternatively, patients with the G allele are more prone to diabetic complications which are mostly caused by hyperglycemia and associated glucotoxicity (28). Second, the AA patients may require lower doses of oral hypoglycemic or even insulin treatment to avoid possible hypoglycemia associated with excessive insulin or OHA dosing. Third, although more population-based studies are required, for *SERPINB1* SNP rs15286 so far, we can consider the G allele as the “bad allele” or the “risk allele.” Diabetic patients who are carriers of the G allele are under risk of poor glycemic control and should be closely monitored for their hyperglycemia. However, clinical trials are warranted to test these hypotheses.

Another interesting finding in this study is the higher HOMA2-β% associated with the AA genotypes in comparison to other genotypes. SerpinB1 has been portrayed as a β-cell-protective hepatokine. Whether the AA genotype may provide enhanced/better protection than other genotypes is a question that warrants further investigations especially in prediabetic patients. It is noteworthy that this is the first study that explores the association of a *SERPINB1* SNP variant with β-cell function in diabetic patients.

Nevertheless, this study faced several limitations that we have been aware of. First, further studies with larger samples representable of the different ethnic populations are warranted. Second, our failure to find various variants of the SNP rs114597282 constrained our capability to further study this SNP. In fact, although this study was limited by its relatively small sample size, our results demonstrate the interplay of *SERPINB1* SNP rs15826 with glycemic control in diabetic patients and shed light on the possible implication of *SERPINB1* gene polymorphism in diabetes pathogenesis, as well as the risk for developing diabetic complications.

In conclusion, to the best of our knowledge, this is the first report to show that the *SERPINB1* gene has the SNP rs15286 variant of all genotypes expressed in the Egyptian population, with the A allele as the minor allele of about 35% of the population. However, these genotypes are not associated with risk to diabetes. The AA genotype of this SNP is associated with an overall better glycemic control and better β-cell function in diabetic patients. The G allele can be considered as the “risk allele” for poor glycemic control in diabetic patients. Conclusively, *SERPINB1* SNP rs15826 can potentially predict glycemic control in diabetic patients and can enhance better treatment options for these patients based on their genotypes. In addition, this study opens the door for further studies to investigate the possible association between other *SERPINB1* gene variants and susceptibility for diabetes and/or diabetic complications in different ethnic populations. Furthermore, further research is required to study the effect of these SNPs on the serum levels of serpinB1, which may explain the better glycemic control associated with various genotypes. Finally, further studies are warranted to further elucidate the clinical impact of rs15286 variants on diabetic patients' treatment regimen in Egyptian as well as other populations.

## Data Availability Statement

The raw data supporting the conclusions of this article will be made available by the authors, without undue reservation, to any qualified researcher.

## Ethics Statement

The studies involving human participants were reviewed and approved by National institute of Diabetes and Endocrinology—General Organization for Teaching Hospitals and Institutes (NIDE – GOTHI). The patients/participants provided their written informed consent to participate in this study.

## Author Contributions

MK and DK conceived and designed the experiments, analyzed the data, and wrote the manuscript. MK, DK, and AA performed the experiments. GH and AA provided and processed the samples. All the authors reviewed and approved the manuscript. All authors contributed to the article and approved the submitted version.

## Conflict of Interest

The authors declare that the research was conducted in the absence of any commercial or financial relationships that could be construed as a potential conflict of interest.
